# Structural basis for PPAR partial or full activation revealed by a novel ligand binding mode

**DOI:** 10.1038/srep34792

**Published:** 2016-10-06

**Authors:** Davide Capelli, Carmen Cerchia, Roberta Montanari, Fulvio Loiodice, Paolo Tortorella, Antonio Laghezza, Laura Cervoni, Giorgio Pochetti, Antonio Lavecchia

**Affiliations:** 1 Istituto di Cristallografia, Consiglio Nazionale delle Ricerche, Via Salaria Km. 29, 300, 00015 Monterotondo Stazione, Roma, Italy; 2 Dipartimento di Farmacia, Università degli Studi di Napoli, Via Montesano 49, 80131 Napoli, Italy; 3 Dipartimento di Farmacia-Scienze del Farmaco, Università degli Studi di Bari “Aldo Moro”, Via E.Orabona 4, 70126 Bari, Italy; 4 Dipartimento di Scienze Biochimiche “A. Rossi Fanelli”, Università di Roma “La Sapienza”, Piazzale A. Moro 5, 00185 Roma, Italy

## Abstract

The peroxisome proliferator-activated receptors (PPARs) are nuclear receptors involved in the regulation of the metabolic homeostasis and therefore represent valuable therapeutic targets for the treatment of metabolic diseases. The development of more balanced drugs interacting with PPARs, devoid of the side-effects showed by the currently marketed PPARγ full agonists, is considered the major challenge for the pharmaceutical companies. Here we present a structure-based virtual screening approach that let us identify a novel PPAR pan-agonist with a very attractive activity profile and its crystal structure in the complex with PPARα and PPARγ, respectively. In PPARα this ligand occupies a new pocket whose filling is allowed by the ligand-induced switching of the F273 side chain from a closed to an open conformation. The comparison between this pocket and the corresponding cavity in PPARγ provides a rationale for the different activation of the ligand towards PPARα and PPARγ, suggesting a novel basis for ligand design.

The peroxisome proliferator-activated receptors (PPARs) are members of the II class of nuclear receptors (NRs) superfamily^1^ and play a crucial role in the regulation of metabolic homeostasis. They can induce or repress genes involved in adipogenesis, lipid and glucose metabolism, energy balance, and inflammation. PPARs dynamically shuttle between nucleus and cytoplasm, although they constitutively and predominantly appear in nucleus^2^. The nuclear-cytoplasmic shuttling of PPARs is regulated by respective PPAR ligands^2^. After ligation with their agonists, PPARs heterodimerize with retinoid X receptor (RXR); this complex recognizes specific DNA sequence elements, termed peroxisome proliferator response element (PPRE), in promoters of target genes. The transcriptional activity of PPARs is finely regulated by co-activators or co-repressors, which modulate signaling and interaction with the basal transcription machinery^3^. In the absence of ligands (ligand-independent repression), PPARs bind the promoters of their target genes and repress transcription by recruiting co-repressor complexes^4^ (e.g., NCoR and SMRT). Upon ligand activation (ligand-dependent transactivation), PPARs undergo conformational changes that provoke the displacement of co-repressors and recruitment of co-activators such as p300/CBP and p160, inducing transcription^3^. In contrast to transcriptional activation and repression, there is an additional mechanism defined “transrepression” that involves gene repression in a ligand-dependent manner, interfering with other signal transduction pathways, through protein-protein interactions with NFkB, AP1 and STAT^5^,^6^,^7^. Transrepression does not involve binding to PPREs but is attained through the recruitment and stabilization of co-repressor complexes on the promoters of pro-inflammatory genes. This mechanism might explain the anti-inflammatory properties of PPARs^5^,^6^,^7^,^8^. To date, there are three known subtypes of PPAR receptors: α, γ, and δ. PPARα is expressed in tissues with a high rate of fatty acid catabolism and modulates lipid metabolism and inflammation^9^. PPARγ is predominant in adipose tissue where it induces lipogenesis and fat storage, and in skeletal muscle, where it improves insulin sensitivity^10^, whereas the PPARδ subtype is ubiquitously expressed but remains the less understood PPAR subtype and would benefit from further development of pharmacological tools^11^. However, recent studies have identified the role of PPARδ in cholesterol metabolism, adipocyte differentiation, neuronal function and colon cancer^12^. PPARs, therefore, represent valuable therapeutic targets and PPAR agonists have for many years represented a promising approach for the treatment of type 2 diabetes and associated metabolic diseases, including obesity, hypertension, and dyslipidemia^11^. PPARα agonists, represented by fibrates (e.g., fenofibrate and gemfibrozil), are clinically used for the treatment of dyslipidemia. The thiazolidinediones (TZDs) or glitazones (e.g., rosiglitazone and pioglitazone) are considered as specific ligands for PPARγ and are being used for treating hyperglycemia in patients with type 2 diabetes. However, side effects including weight gain, edema, congestive heart failure, and the recently reported increased risk of bone fracture following treatment with rosiglitazone or pioglitazone are major undesired effects associated with the use of PPARγ full agonists^13^. Due to these effects, the use of rosiglitazone was restricted or suspended by some regulatory agencies around the world^14^. For this reason, an alternative approach for the treatment of metabolic disorders is represented by the development of partial PPARγ agonists or selective PPARγ modulators. Partial agonists are defined as weak activators of PPARγ that elicit the same activation pattern and show linked dose–response curves with lower transactivation potential compared to full agonists. They might induce alternative receptor conformations and thus recruit different co-activators, resulting in distinct transcriptional effects compared to classical glitazones^11^. Structural studies indicated that partial agonists do not interact directly with helix 12 (H12), as do TZDs and other full agonists, but differentially stabilize other regions of the binding pocket, as the helix 3 (H3) and β-sheet regions of PPARγ LBD^15^. In particular, they form a combination of H-bonds to the backbone amide of S342 and electrostatic interactions with R288, as well as extensive van der Waals interactions with I341 of the β-sheet and C285 of H3^16^. In recent years a new antidiabetic mechanism has been proposed depending upon the inhibition of PPARγ S245 phosphorylation (PPARγ2 residue S273) by cyclin-dependent kinase 5 (Cdk5)^17^. This inhibition maintains the transcription of several insulin-responsive genes, as adiponectin and adipsin^17^. Interestingly, obesity and other pro-inflammatory signals induce the phosphorylation of PPARγ at S245, explaining why obese people also develop insulin resistance. Given that the Cdk5 recognition site extends into the first β-strand of PPARγ, structural stabilization of the β-sheet region, elicited by partial agonists, presumably renders the residue S245 less accessible to the kinase, protecting the receptor from phosphorylation^17^, an event that correlates well with glucose-lowering effects. This might explain how PPARγ partial agonists can exhibit similar or higher antidiabetic effects than those of TZDs and other full agonists.

A further research direction under consideration is to explore the therapeutic potential of PPAR pan-agonists that would combine the agonistic activities toward PPARγ, PPARα, and PPARδ in a single ligand but with a balanced activation profile; these compounds may prove to be the ultimate combination of PPAR activities for treatment of type 2 diabetes and its further complications. Indeed, PPAR pan-agonists would regroup the beneficial effects of the three PPAR subtypes by normalizing insulin resistance, plasma lipids, and adiposity^11^. Several ligand- and/or structure-based computational methods have been used to target these nuclear receptors^18^,^19^,^20^,^21^,^22^. Previous work by our group has shown the beneficial application of ligand- and structure-based virtual screening approaches to novel hit discovery in many protein targets^23^,^24^,^25^,^26^,^27^. In the present study, we performed a structure-based virtual screening (SBVS) approach against PPARγ employing a combination of docking and structure interaction fingerprints (SIFt) based prioritization to discover novel scaffolds for PPARγ partial agonists. Among the 44 compounds identified as putative (virtual) hits, five were confirmed as PPARγ partial agonists through the use of PPAR-Gal4 transactivation assay. Importantly, PPAR subtype selectivity studies identified four dual targeting PPARα/γ agents and one compound as a PPARα specific scaffold. After an extensive filtering of the results and the purchasing and testing of selected compounds, we identified a novel PPAR pan-agonist (compound **56**, also termed AL29-26), with a distinct and balanced activation profile. To gain more insights into the molecular mechanism of PPAR partial or full activation, we solved the crystal structures of AL29-26 bound to PPARα and PPARγ, respectively.

## Results and Discussion

### Structure-based virtual screening

**Figure 1:**
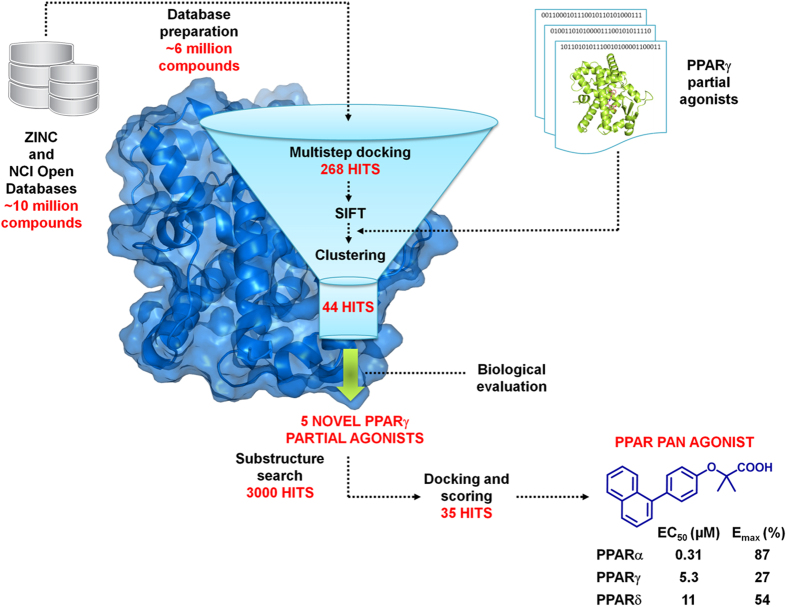
Flow chart of the SBVS strategy implemented in this work.

We performed a SBVS study for the computer-aided discovery of novel PPARγ partial agonists using a protocol that combines a docking scoring function with a SIFt based prioritization. Figure 1 gives an overview of the VS steps.

**Figure 2:**
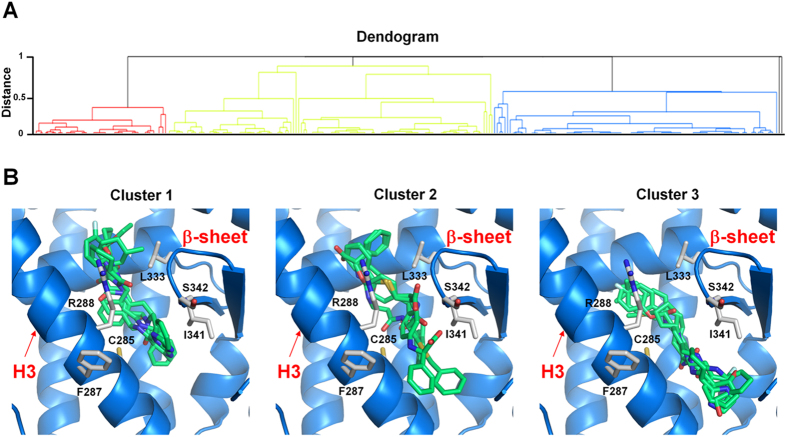
Dendrogram derived by clustering SIFts. (**A**) Dendrogram derived by agglomerative hierarchical clustering of SIFt of PPARγ partial agonists and VS hits. Tanimoto similarity coefficient was used to calculate similarity between the SIFts. (**B**) Binding mode of VS hits belonging to cluster 1 (left), cluster 2 (middle) and cluster 3 (right). Some representatives of structures are shown.

The 3D structure of PPARγ LBD in complex with the partial agonist nTZDpa (PDB: 2Q5S)^16^ was chosen to virtually screen a nonredundant selection of compounds from the Open NCI and ZINC databases^28^. PPAR ligand-like library was built according to a protocol described in the Experimental Procedures and was subsequently used for SBVS with the Glide software^29^,^30^,^31^. In order to select promising compounds on the basis of their ability to form favorable interactions with PPARγ LBD, the 262 top-ranked structures obtained in the previous step were prioritized by the SIFt method^32^,^33^. Since the large binding cavity of PPARγ can accommodate different types of ligands, many of which could not be true binders, a knowledge-based approach can bring additional information to improve success in VS. In fact, it was suggested that scoring should derive from knowledge-based structural comparisons in which binding interactions are considered^34^. Here, SIFTs were used to filter VS hits using knowledge of the co-crystallized ligand binding mode of PPARγ partial agonists. To this aim, a panel of 25 X-ray crystal structures of PPARγ in complex with partial agonists displaying high potency (EC_50_ in low micromolar range) was catalogued from the RCSB protein data bank (PDB)^35^ as reference binding modes (Table S1). SIFTs for 262 VS hits were calculated through the Interaction Fingerprints script in the Maestro interface (Schrödinger) and then clustered using an agglomerative hierarchical clustering approach^36^ and the Tanimoto coefficient (Tc) as the quantitative measure of bit string similarity. The dendrogram derived by clustering SIFts (Fig. 2) revealed three major clusters, each of which represents a distinct binding pattern in the protein-ligand complexes.

Cluster 2 contains VS hits similar to the reference X-ray crystal structures in the pocket between H3 and the β-sheet region of PPARγ. Clusters 1 and 3 are similar in position but represent distinct binding modes that result in dissimilar interactions with the pocket between H3 and the β-sheet of the receptor. Figure 2B shows the bound conformation of some VS hits belonging to cluster 2 strongly mimicking the observed conformation of partial agonists in the PPARγ LBD and able to form H-bonds to the backbone atoms of the β-sheet. Most of the hits found in clusters 1 and 3 (Fig. 2B) are located also in the binding pocket between H3 and the β-sheet region of PPARγ and occupy the same spatial position as the VS hits of cluster 2; however, these compounds are placed slightly higher or lower in the binding pocket and do not form H-bonding interactions with the β-sheet of the protein. In order to prioritize potential hits for biological testing through visual inspection of the clusters and compound binding mode, the following criteria were considered: i) compounds should be from cluster 2, ii) the degree of ligand surface complementarity with the binding pocket; iii) the quality of the overall binding conformation; iv) the formation of a H-bond with S342 and/or R288. This strategy facilitated the selection of a smaller set of 51 compounds, which were screened by FAF-Drugs3 (http://fafdrugs3.mti.univ-paris-diderot.fr) tool^37^ for substructural features that appear in frequent hitters in several biochemical HTS. Finally, 44 molecules were purchased or requested from the NCI Developmental Therapeutics Program (DTP) (Table S2) for biological evaluation.

### Biological Testing of Selected VS Hits

The selected 44 compounds were biologically tested for PPAR activity using a cell-based hPPAR-LBD transactivation assay for each of the three PPAR subtypes in order to determine the selectivity of the compounds in activating PPARα, PPARγ, and PPARδ. For this purpose, Gal4-PPAR chimeric receptors were expressed in transiently transfected HepG2 cells according to a previously reported procedure^38^,^39^,^40^,^41^. The results were compared with those obtained with Wy-14,643, rosiglitazone, and L-165,041 used as reference compounds of PPARα, PPARγ, and PPARδ, respectively. Five compounds, namely **6**, **13**, **33**, **38**, and **40** were found to act as PPAR ligands and exhibited EC_50_ in the low micromolar range (Table S2).

With the aim of exploring chemical space and investigate structure-activity relationships (SARs), we searched for derivatives matching the pharmacophore fragment (bolded in Fig. 1), common to compounds **6**, **13**, **33**, **38**, and **40** and presumed to be the bearer of the PPAR activity of such compounds. Substructure searches were conducted by means of the Canvas 1.9 software (Schrödinger) on the NCI Open and ZINC databases. To expand the number of substructures considered, we also included structurally related queries that maintained the key pharmacophore. The results of each of these searches were submitted to docking, and through a series of filters designed to reduce docking false positives, 25 compounds were selected for purchase (Table S3). After testing, we identified four PPAR ligands (**49**, **56**, **59** and **69** in Table 1). Specifically, compounds **49** and **69** turned out to be selective partial agonists on PPARγ subtype, while compound **59** was a selective PPARα partial agonist. Interestingly, the naphthalenic derivative **56** (hereinafter termed AL29-26) showed a very attractive PPAR pan-agonist activity profile: potent full agonist on PPARα and partial agonist on PPARγ and δ subtypes. See Figs S1 and S2 of Supporting Information for experimental data related to fold induction and dose-response curves in transactivation assay.

### Structure determination

**Figure 3:**
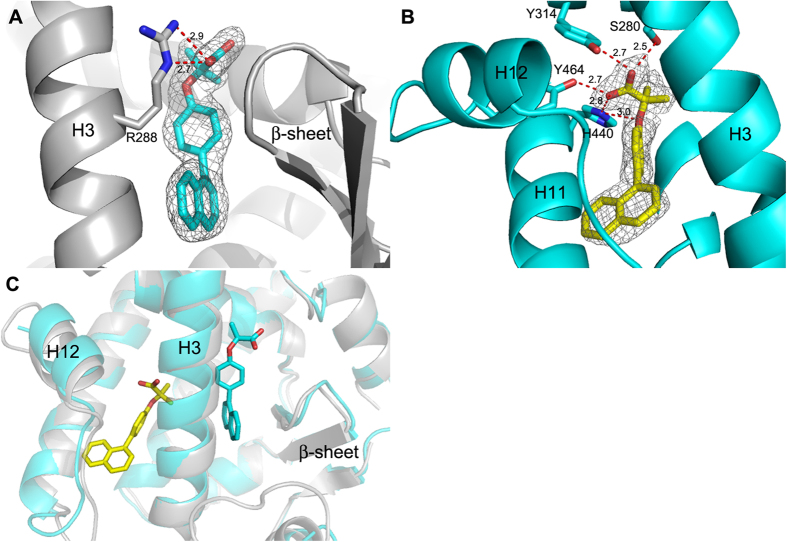
Ligand binding to PPARα and PPARγ. (**A**) Binding mode of AL29-26 (cyan) in the LBD of PPARγ (grey). (**B**) Binding mode of AL29-26 (yellow) in the LBD of PPARα (cyan); 2Fo-Fc omit maps are shown in mesh and contoured at 1σ. (**C**) Superposition of PPARα/AL29-26 (ligand yellow, protein cyan) and PPARγ/AL29-26 (ligand cyan, protein gray) crystal structures.

X-ray diffraction data were collected for the complexes PPARα/AL29-26, PPARγ/AL29-26 and PPARδ/AL29-26 to provide an explanation at the molecular level for the different behaviour of AL29-26, as partial or full agonist, towards the three PPAR subtypes. The crystal structures of PPARα and PPARγ complexes were solved and the ligand could be unambiguously modelled in the electron density maps (Fig. 3A–C). The crystallographic statistics are shown in Table 2. Diffraction data were also collected for PPARδ/AL29-26 at low resolution (4–5 Å) but no interpretable electron density maps could be obtained.

### PPARγ/AL29-26 structure

As known, the apo-form of PPARγ crystallizes as a dimer, where the molecule A of the asymmetric unit has its activation-function 2 helix (H12) in the active conformation and the molecule B in the inactive conformation, due to the crystal packing. AL29-26 was very easily modelled in the density of the molecule B and its binding mode in the LBD of PPARγ is shown in Fig. 3A. A second molecule of the ligand could be also fitted in molecule A of the dimer with a similar binding mode. The protein-ligand interactions observed in chains A and B were basically the same, consequently, the two complexes of the asymmetric unit will be described hereafter as a generic model.

### New position of the carboxylate in the region of PPARγ partial agonists

**Figure 4:**
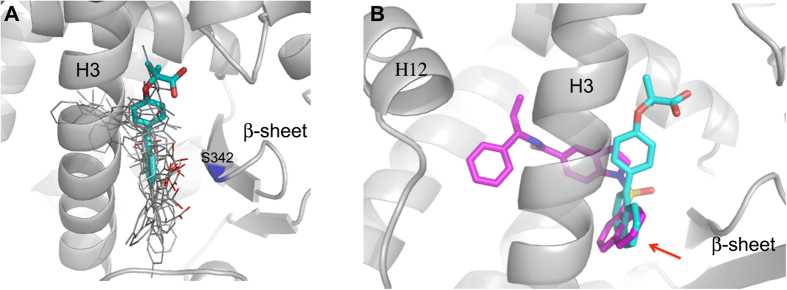
Comparison of PPARγ complexes. (**A**) Superposition of PPARγ complexes (gray) with known partial agonists (pdb codes: 3D6D, 4PVU, 4PWL, 4JL4, 4JAZ, 4E4K, 2Q5P, 2Q6S, 2Q5S, 4E4Q, 5F9B) onto the PPARγ complex with AL29-26 (cyan). The carboxylate groups are depicted in red, the residue S342 in blue. (**B**) Superposition of SR2067 (magenta) (pdb code 4R06) onto the PPARγ complex with AL29-26 (cyan). The red arrow indicates the naphthalene groups of the two ligands.

Remarkably, the ligand occupies the canonical region of PPARγ partial agonists, between helix 3 and the β-sheet, but its position is significantly different from that of all the partial agonists known in literature (Figs 4A and S3A).

Usually, all the PPARγ partial agonists form a more or less efficient H-bond interaction through their carboxylate group with the NH of S342, belonging to inner strand of the β-sheet. On the contrary, the carboxylate of AL29-26 is significantly shifted along the axis of H3 towards the helix 5, forming two H-bonds with the side chain of R288 through one of its oxygens (2.7 and 2.9 Å) (Fig. 3A). The other carboxylate oxygen makes H bonds (2.7 Å) with two water molecules bridged to CO of L228 on the loop H1-H2a (2.8 Å) and to CO of M329 on the helix 5 (2.9 Å), respectively.

One of the two methyl groups of the ligand makes vdW interactions with the methyls of the A292 and M329 side chains (3.8 and 4.3 Å, respectively), the other one with those of L330 and L333 (4.2 and 4.1 Å, respectively). The single aromatic ring is sandwiched between the R288 and L330 side chains. The naphthalene ring is positioned between I341 and C285 side chains, facing the NH S342, on the β-sheet, usually H-bound to the carboxylate group of standard partial agonists. The naphthalene system of the ligand is almost perpendicular to the plane of the single aromatic ring.

### PPARα/AL29-26 structure

In the complex PPARα/AL29-26, the polar head of AL29-26 is engaged in a very efficient network of H-bonds with the side chains of Y464 (2.7 Å) on the H12 helix, crucial for regulation of the co-activator recruitment, Y314 (2.7 Å), H440 (2.8 Å) and with the OH of S280 (2.5 Å) (Fig. 3B). These standard polar interactions are shared by all the agonists complexed to PPARα reported in the PDB. The two methyl groups of the ligand form vdW contacts with M355, F318, H440 and C276, in the upper part of the binding pocket.

### The ligand occupies a new pocket in the PPARα LBD

**Figure 5:**
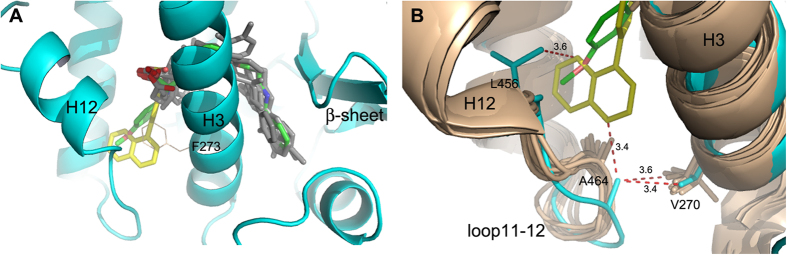
Comparison of PPARα complexes. (**A**) Superposition of PPARα complexes (gray) with known partial agonists (pdb codes: 2REW, 4BCR, 1K7L, 3SP6, 3FEI, 2GTK, 3G8I, 3ET1, 3KDT, 1I7G) onto the PPARα complex with AL29-26 (ligand yellow, protein cyan). The ligand BMS-631707 (PDB code 2REW) is shown in green. The “closed” (trans) conformation of F273 side-chain is also shown (gray). (**B**) New conformation of the loop 11–12 in the PPARα/AL29-26 complex: superposition of the loops 11–12 of known PPARα structures (light-brown) (same pdb codes of Fig. 5A) with that of PPARα/AL29-26 (ligand yellow, protein cyan). The ligand BMS-631707 (PDB code 2REW) is shown in green. Additional vdW interactions realized by AL29-26 are shown as red dashed lines.

The rigid and bulky aromatic groups of the ligand occupy a new region of the PPARα hydrophobic pocket, between H3 and H11, never occupied by other known PPARα agonists (Figs 5A and S4A), with the only exception of the ligand BMS-631707 (PDB accession code 2REW)^42^.

**Figure 6:**
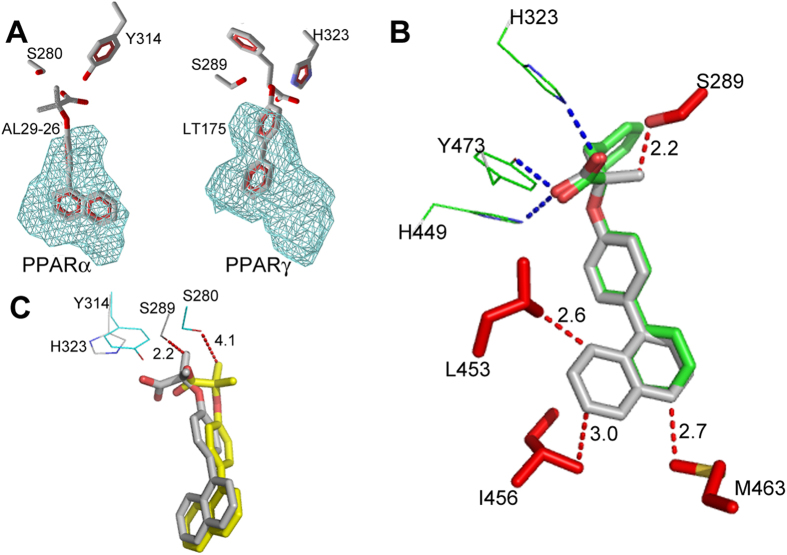
New PPARα and PPARγ hydrophobic pockets. (**A**) New PPARα (left) and PPARγ (right) hydrophobic pockets allowed by the ligand-induced switching of the F273 side-chain (F282 in PPARγ). (**B**) Modelled AL29-26 (gray) onto LT175 (green) (pdb code 3B3K) in the complex with PPARγ. Residues belonging to the “diphenyl pocket” are shown in red (vdW interactions with AL29-26 as red dashed lines). The residues interacting with the carboxylate group are shown in green (H-bonds are shown as blue dashed lines). (**C**) Superposition of PPARγ-modelled AL29-26 (gray) onto the PPARα/AL29-26 complex (the ligand is shown in yellow). Two representative residues of PPARγ are shown in gray, the corresponding residues of PPARα in cyan.

The opening of this usually inaccessible region is allowed by the ligand-induced switching of the F273 from the generally adopted extended to the folded g* conformation (χ^1^ = −67°). As a consequence, the aromatic moiety of the ligand occupies the position usually occupied by the aromatic ring of F273, that acts as a gate-keeper for the accommodation of rigid and bulky substituents at the carbon atom linked to the carboxylate group. The above mentioned ligand BMS-631707, containing the conformationally constrained azetidinone ring linked to the carboxylate, behaves in the same way replacing the side chain of F273, forced to assume a gauche conformation. A similar situation was also observed in the complex of PPARγ with LT175 (PDB code 3B3K) where the rigid and straight diphenyl group of the ligand induced the flipping-out of the corresponding F282 side chain, towards the “benzophenone pocket”, making available a new region of the LBD, the so-called “diphenyl pocket”^43^. In that case, it was observed that the diphenyl pocket is L-shaped and the diphenyl group of LT175 occupied only the first branch (Fig. 6A). In PPARα the similar, but more spherical, new region of the LBD is totally filled, by the single aromatic ring of AL29-26, that occupies the first branch, whereas the naphthalene rings fill the second branch of the cavity (Fig. 6A).

### AL29-26 interactions in the new pocket promote a local stabilization of PPARα LBD and AF-2 region

The accommodation of the ligand in this new region induces a significant conformational change of the loop 11–12 that forms the edges of the pocket with H3 and H11. It is worth noting that in all the known structures of PPARα complexes this loop always assumes a different and ordered conformation in which the residue A454 would make a steric clash with the bulky naphthalene rings of AL29-26. Consequently, the ligand provokes a rearrangement of the loop 11–12 whose new conformation, closer to H3, is strongly stabilized by a vdW interaction between A454 and the two methyls of V270, on helix 3 (3.4 and 3.6 Å) (Figs 5B and S4B). A further stabilization is achieved through vdW interactions of the methyl groups of A454 and L456 with carbon atoms of the naphthalene ring system (3.4 and 3.6 Å, respectively). This novel and distinct interaction between H3 and the loop 11–12, indirectly, also contributes to further stabilize the active conformation of H12.

### Specificity of the new cavity: a steric clash with S289 prevents AL29-26 to occupy the diphenyl pocket of PPARγ determining its partial agonist properties

We investigated the reason why AL29-26 in PPARγ complex doesn’t occupy the diphenyl pocket, preferring to fill the region of partial agonists. AL29-26 was modelled in the PPARγ diphenyl pocket taking into account the different hydrogen-bonds network realized by the carboxylate group with respect to PPARα, for the presence of H323 in place of Y314. For this purpose we used the structure of the PPARγ/LT175 complex as template, given the similarities between the two ligands. AL29-26 could occupy the diphenyl pocket, entirely filling the second branch of the cavity even though with very short vdW distances with some residues of the protein (2.7 Å with M463, 2.6 Å with L453, 3.0 Å with I456 side chains) but in this position one of its methyls would make a steric clash with the OH of S289 (2.2 Å), preventing its H-bonding with the carboxylate group and perturbing in this way the efficient H-bond network of the ligand (Fig. 6B).

Conversely, in the PPARα/AL29-26 complex the new hydrophobic pocket has more space to accommodate the ligand because of the shorter A454 in place of M463 of PPARγ, the shorter V444 instead of L453, and the side chain of I447, less close to the ligand with respect to the corresponding I456. The global volume of the two cavities calculated by the software Molegro^44^ appears to be very similar (200.7 versus 198.1 Å^3^ for PPARα and PPARγ, respectively), but unlike the L-shaped PPARγ diphenyl pocket, the PPARα cavity has an almost spherical shape that better allows the accommodation of a bulkier ligand (Fig. 6A). In this situation, the two methyls of the ligand have no clashes with the side chain of S280 (S289 in PPARγ), due to the shifted positioning of the ligand in the pocket caused by the presence of the bulkier Y314 on helix 12, with respect to PPARγ H323 (Fig. 6C).

At this regard, we noticed that in the molecule A of PPARγ, whose H12 is in its active conformation, there is weak electron density in correspondence of the side chain of F282 in its closed conformation, and some residual Fo-Fc electron density is visible in the region corresponding to its “flipped-out” conformation; moreover, there is some poor, not easily interpretable, additional density in the diphenyl pocket that cannot be merely attributed to water molecules and that let hypothesize a small percentage of occupation of this region by a second molecule of ligand with lower affinity. This could also be a consequence of the soaking method used to bind AL29-26 in the crystal of apo-PPARγ, whereas the ligand is in large excess with respect to the protein. However, this event is not visible in the molecule B of PPARγ where H12, in its inactive conformation, cannot interact with the ligand. In this case the side chain of F282 assumes unambiguously the extended conformation, blocking the entrance of the new pocket. In conclusion, AL29-26 in the PPARγ complex prefers to occupy the region facing the β-sheet, behaving as a partial agonist. It could be hypothesized that the substitution of one of the two methyl groups of AL29-26 with a H atom could relieve the steric clash with S289 of PPARγ changing the pharmacological character of this ligand and turning it in a more potent agonist.

### PPAR’s LBD stabilization upon ligand binding

Differential scanning microcalorimetry (DSC) experiments were performed to confirm the PPAR’s LBD stabilization upon ligand binding. Figure S5A,B show the DSC curves of PPARγ. After the main transition at 46.6 °C, the excess heat capacity does not remain constant as expected for a completely unfolded state, but increases with increasing temperature, resulting in a second broad heat absorption peak in the temperature range 55–80 °C. A broad diffuse heat absorption peak has been observed for unfolding of the molten globule or molten globular-like state of many proteins. In presence of AL29-26 (Fig. S5C,D) the main transition is at 47.5 °C and the second broad heat absorption peak is lower. This result shows that the presence of the ligand establishes a conformational change in PPARγ. Figure S6A,B show the DSC curves of PPARα. The main transition is one two-state transition at 41.9 °C. In the presence of the ligand (Fig. S6C,D) the transition occurs at 42.6 °C with deconvolution of the excess heat capacity function into two two-state transitions (37.6 °C and 43.5 °C). A DSC experiment was also performed on PPARδ but produced thermograms of bad quality, difficult to interpret (data not shown). In conclusion, the experimental data of PPARγ and PPARα showed only a slight shift of the denaturation peak upon ligand binding (about 1 °C). A previous DSC experiment with bulky ureidofibrates PPARγ ligands^42^ showed shifts of 2–5 °C, indicating that bulkier compounds may better stabilize the dynamics of the LBD.

### Concluding remarks

A SBVS approach let us identify a novel PPAR pan-agonist with an interesting activation profile. The ligand AL29-26 has been shown to be a potent full agonist on PPARα, and a partial agonist on PPARγ and δ subtypes. To date, the data show that an appropriate dual PPARα/γ activation results in improved metabolic profile. However, an over-activation of PPARγ can lead to serious side effects including weight gain and steatosis, for this reason PPARγ partial agonists are more desirable. On the other hand, a strong activation of PPARα is also advantageous because improves dyslipidemia, lowering plasma triglycerides and increasing HDL cholesterol levels. Therefore, the search for the optimal pharmacological profile of a ligand plays an important role and for this purpose it is required the understanding, at molecular level, of the mechanism by which the ligand behaves as full or partial agonist on PPARs. In this paper we provided a rationale for the difference of ligand activity toward PPARα and PPARγ subtypes. We demonstrated that the ligand AL29-26 could yet occupy the diphenyl pocket in PPARγ, despite its narrower shape with respect to that of PPARα, but in this position one of its methyls, would make a strong steric clash with the OH of S289, preventing its H-bonding with the carboxylate group and perturbing in this way the efficient H-bond network of the ligand. For this reason, AL29-26 in PPARγ prefers to occupy the typical region of partial agonists, facing the β-sheet and not interacting with H12, and assuming in this way a character of partial agonist. One can speculate that a new ligand with only one or no methyl groups, in alpha position to the carboxylate group, could probably occupy the PPARγ diphenyl pocket behaving as a more potent agonist. This issue will be addressed in the next work. Differently, the shifted position of the ligand in the PPARα new pocket, caused by the presence of the bulkier Y314 side chain with respect to PPARγ H323, allows to avoid the clash with the side chain of S280 (the equivalent of S289 in PPARγ) enabling the occupation of the larger and more spherical-shaped hydrophobic cavity without being perturbed the H-bond network of the carboxylate. The discovery of this new cavity of PPARα, analogue to the PPARγ diphenyl pocket, whose accessibility is regulated by the side chain of the gate-keeper F273, was never observed before and its structural characterization opens new opportunities to rational design of more balanced PPAR modulators. Moreover, we confirmed in this work the many possibilities used by PPARs to modulate the stabilization of the activation function 2 (AF-2) region through a subtle mechanism of molecular cross-talk, mediated by the ligand, among different regions of the protein. It is known the unique mode of binding to PPARα of the ligand WY14643^45^ (pdb code 4BCR) that revealed a new pattern of nuclear receptor ligand recognition in which a second molecule of ligand is involved in the interaction with the protein, providing additional stabilization to the AF-2 region. This molecule strongly stabilizes the highly mobile ω-loop by the formation of a charge cluster that involves the ligand itself, the helix 3 and the H11–12 loop, providing in this way a more subtle stabilization of H12. At the same way, in this work we showed that the bulky AL29-26, occupying a new hydrophobic pocket, perturbs the PPARα standard conformation of the loop 11–12 forcing it to a different and stable conformation in which A454 of the loop strongly interacts with V270 on H3. This novel interaction between H3 and the loop 11–12, mediated by the ligand, also contributes to further stabilize the active conformation of H12. Similar mechanisms have been proposed to explain actions of PPARγ partial agonists, that activate H12 to a lesser extent stabilizing H3 and the β-sheets and/or modifying the dynamics of the flexible ω-loop^16^. The structure of the complex PPARγ/AL29-26 showed a different arrangement of the ligand in the region facing the β-sheet, shifted along the axis of H3, where the H-bond with NH of S342 is lost and there is a strong interaction with the side chain of R288. It would be interesting to ascertain whether this unusual position of a partial agonist could be associated to a different stabilization of the β-sheet and, consequently, to a different degree of inhibition of the S245 phosphorylation with respect to other partial agonists. At this regard, it has been recently published the crystal structure of PPARγ with the ligand SR2067 (pdb code 4R06) that interacts with the β-sheet exclusively via hydrophobic interactions mediated through a naphthalene group, revealing a unique kinetic and structural signature for PPARγ partial agonism^46^. The superposition of this structure with PPARγ/AL29-26 shows an equivalent position of the naphthalene groups of the two ligands, in front of the β-sheet (Figs 4B and S3B).

In conclusion, we discovered a new mode of ligand binding to PPARα and provided a structural basis of ligand design, offering clues for the development of drugs with a more balanced activation profile for the treatment of patients suffering from dyslipidemia and type 2 diabetes.

## Experimental Procedures

### PPAR ligand-like library preparation and SBVS protocol

The PPAR ligand-like library for SBVS was obtained by merging commercial screening collections (Ambinter, Maybridge, Chembridge, Asinex, TimTec, Innovapharm, listed on the ZINC Web site) and the NCI Open Database (http://dtp.cancer.gov/), and then by the filtering of duplicates, using Biovia’s Pipeline Pilot (version 9.2, San Diego, CA). In the libraries, all compounds containing inorganic atoms were removed prior to any processing. Then, all the structures of the compounds were chemically standardized (including adding hydrogen atoms, ionizing at the pH range from 5.1 to 9.1, and generating stereoisomers and valid single 3D conformers) by means of the LigPrep module in Maestro (version 3.5, Schrödinger).

The X-ray coordinates of PPARγ LBD in complex with the partial agonist nTZDpa (PDB: 2Q5S)^16^ were used as the structural template for SBVS with molecular docking approaches. The template was manipulated with the “Protein Preparation Wizard” workflow in Maestro. The main manipulations are removing all water molecules, protonation, and optimization based on OPLS_2005 force field. Then, a docking grid was generated using the “Receptor Grid Generation” module of Maestro. The grid encloses a box centered on the native ligand nTZDpa with a dimension of 20 × 20 × 20 (*x* × *y* × *z*, Å). The scaling factor of 0.8 was set for van der Waals radii of receptor atoms with a partial atomic charge less than 0.15.

The virtual screening workflow of Glide was employed to screen the PPAR ligand-like library. It performed the docking mainly in three phases, namely HTVS (high-throughput virtual screening), SP (standard-precision) and XP (extra-precision). Using HTVS, we screened our library and reduced the intermediate conformations. Successful compounds (2%) from HTVS were further assessed in SP docking for reliable docking of the screened compounds with high accuracy. To eliminate false-positive results, the best 20% of thriving compounds from SP docking were further incorporated for XP docking mode using advanced scoring.

The SIFt method^32^,^33^ was used to identify amino acids that interact with the complexed ligand. SIFts were calculated by using the user interface (GUI) script implemented in Schrodinger’s Small Molecule Drug Discovery Suite (interaction_fingerprints.py). The results were stored in a 1D binary string, in which a 9-bit pattern was used to describe the interaction type: any contact, backbone, side chain, polar, aromatic, hydrophobic interaction, H-bond donor/acceptor, and charged.

To analyze and reorganize each library of poses, we applied an agglomerative hierarchical clustering^36^ in Canvas (version 2.6, Schrödinger), using Tanimoto coefficients^47^ as the similarity measurement. Clusters of protein-ligand complex structures were manually selected based on the dendrogram of their SIFTs.

### Substructure Search

The NCI Open Database, processed as described in the previous section, and ZINC drug-like subset were exposed to a substructure search using Canvas; only those compounds satisfying the pharmacophore query in Fig. 1 were kept. To expand the number of substructures considered, we also included structurally related queries that maintained the key pharmacophore. The results of each of these substructure searches were subsequently docked against PPARγ LBD, and through a series of filters designed to reduce docking false positives, 25 compounds were selected for purchase and biological testing.

### Biological methods

Reference compounds, the medium, and other cell culture reagents were purchased from Sigma-Aldrich (Milan, Italy).

#### Plasmids

The expression vectors expressing the chimeric receptor containing the yeast Gal4-DNA binding domain fused to the human PPARα, PPARγ, or PPARδ ligand binding domain (LBD) and the reporter plasmid for these Gal4 chimeric receptors (pGal5TKpGL3) containing five repeats of the Gal4 response elements upstream of a minimal thymidine kinase promoter that is adjacent to the luciferase gene were described previously^48^.

#### Cell culture and transfections

Human hepatoblastoma cell line HepG2 (Interlab Cell Line Collection, Genoa, Italy) was cultured in minimum essential medium (*2*) containing 10% heat-inactivated fetal bovine serum, 100 U of penicillin G mL^−1^, and 100 μg of streptomycin sulfate mL^−1^ at 37 °C in a humidified atmosphere of 5% CO_2_. For transactivation assays, 10^5^ cells per well were seeded in a 24-well plate and transfections were performed after 24 h with CAPHOS, a calcium phosphate method, according to the manufacturer’s guidelines. Cells were transfected with expression plasmids encoding the fusion protein Gal4-PPARα-LBD, Gal4-PPARγ-LBD or Gal4-PPARδ-LBD (30 ng), pGal5TKpGL3 (100 ng), and pCMVβgal (250 ng). Four hours after transfection, cells were treated for 20 h with the VS ligands and reference compounds in duplicate. As a preliminary assay, all compounds from virtual screening were tested at two concentrations (5 and 25 μM); afterwards, only for ligands showing efficacy higher than 10% a dose-response curve was carried out. Luciferase activity in cell extracts was determined by a luminometer (VICTOR^3^ V Multilabel Plate Reader, PerkinElmer). β-Galactosidase activity was determined using ortho-nitro-phenyl-β-D-galactopyranoside as described previously^49^. All transfection experiments were repeated at least twice.

### PPAR Protein Expression and Purification

PPARγ and PPARα LBDs were expressed N-terminal His-tagged proteins using a pET28 vector and then purified as previously described^50^. Briefly, freshly transformed *E.coli* BL21 DE3 were grown in LB medium with 30 μg of kanamycin/ml at 310 K to an OD of 0.6. The culture was then induced with 0.2 mM isopropyl-β-D-thio-galactopyranoside and further incubated at 291 K for 20 h. Cells were harvested and resuspended in a 20 ml/liter culture of Buffer A (20 mM Tris, 150 mM NaCl, 10% glycerol, 1mM Tris 2-carboxyethylphosphine HCl (TCEP), pH 8) in the presence of protease inhibitors (Complete Mini EDTA-free; Roche Applied Science). Cells were sonicated, and the soluble fraction was isolated by centrifugation (35,000 × g for 45 min). The supernatant was loaded onto a Ni^2+^ -nitrilotriacetic acid column (GE Healthcare) and eluted with a gradient of imidazole 0–500 mM in Buffer A (batch method). The pure protein was identified by SDS PAGE. The protein was then dialyzed over buffer A to remove imidazole, and it was cleaved with thrombin protease (GE Healthcare) (10 units/mg) at room temperature for 2 h. The digested mixture was reloaded onto a Ni^2+^-nitriloacetic acid column to remove His tag and the undigested protein. The flow-through was dialized with buffer B (20mM Tris, 10% glycerol, 1 mM TCEP, pH 8) to remove NaCl and then loaded onto a Q-Sepharose HP column (GE Healthcare) and eluted with a gradient of NaCl 0–500 mM in Buffer B with a BioLogic DuoFlow FPLC system (Bio-Rad Laboratories, Italy). Finally, the proteins were purified by gel-filtration chromatography on a HiLoad Superdex 75 column (GE Healthcare) and eluted with Buffer C (20 mM Tris, 1 mM TCEP, 0.5 mM EDTA, pH 8). The proteins were then concentrated at 8mg/ml using Amicon centrifugal concentrators with a 10 kDa cutoff membrane (Millipore, USA).

### Crystallization and Data Collection

Crystals of apo-PPARγ were obtained by vapor diffusion at 18 °C using a sitting drop made by mixing 2 μL of protein solution with 2 μL of reservoir solution (0.8 M Na Citrate, 0.15M Tris, pH 8.0). The crystals were soaked for several days in a storage solution (1.2 M Na Citrate, 0.15M Tris, pH 8.0) containing the ligand AL29-26 (0.25 mM). The ligand dissolved in DMSO (50 mM) was diluted in the storage solution so that the final concentration of DMSO was 0.5%. The storage solution with glycerol 20% (v/v) was used as cryoprotectant. Crystals (0.2 × 0.2 mm) of PPARγ/AL29-26 belong to the space group *C2* with cell parameters shown in Table 2. Preliminary PPARα co-crystallization trials were performed with a Phoenix liquid-handling robot (Art Robbins) with the ligand AL29-26 in excess 3:1. Crystals (0.7 × 0.2 mm), obtained at the conditions A12 of the Qiagen JCSG-I Core Suite (PEG 3350 20%, 0.2 M Mg Acetate), were freezed using the mother solution with PEG3350 35% (v/v) as cryoprotectant. The crystals of PPARα/AL29-26 belong to the space group *P4*_*1*_*2*_*1*_*2* with cell parameters shown in Table 2.

### Structure Determination and Refinement

X-ray data set were collected at 100 K under a nitrogen stream using sinchrotron radiation (beamline ID 23-2 at ESRF, Grenoble, France). The collected data were processed using the programs MOSFLM and SCALA^51^. Structure solution was performed with AMoRe^52^, using the coordinates of PPARγ/LT175R^43^ (PDB code 3D6D) as the starting model for PPARγ and PPARα/APHM13^53^ (PDB code 3VI8) for PPARα. The coordinates were then refined with CNS^54^. All data between 50.00 and 2.0 Å (1.83 Å for PPARα/AL29-26) were included for PPARγ/AL29-26 belonging to *C2* space group (*P4*_*1*_*2*_*1*_*2* for PPARα/AL29-26). A final step of refinement was performed with the software Phenix^55^. The statistics of crystallographic data for both complex structures and refinement are summarized in Table 2.

### Differential Scanning Calorimetry

DSC experiments were performed with a MicroCal VP-DSC microcalorimeter (MicroCalInc., Northampton, MA, USA). The samples were dialyzed against the Hepes buffer (Hepes 20 mM, pH 8.0, TCEP 1 mM) and gently degassed before scanning. The protein concentration was 10 μM, and the ligand concentration was 20 μM. The concentration of PPAR was determined spectrophotometrically using the extinction coefficient at 280 nm. The reference cell was filled with the same solvent mixture as that used for the sample, but lacking the protein. The experiment was performed ranging from 10 to 100 °C, and the heating rate was 1 °C·min^−1^. Thermograms were corrected by subtracting the instrumental baseline, obtained with both cells filled with the same solvent, and normalized for protein concentration. When a post-transitional baseline could be determined, a progress baseline was subtracted; otherwise, a straight line connecting the initial and the final temperature of the overall transition was used. T_m_ (temperature of maximum heat capacity) and ΔH (heat reaction) were calculated using the Origin 7.0 software provided by MicroCal.

## Additional Information

**Accession Codes**: The coordinates and structure factors for the PPARα /AL29-26 and PPARγ /AL29-26 structures described here have been deposited in the PDB under accession numbers 5HYK and 5HZC, respectively. Details are listed in Table 2.

**How to cite this article**: Capelli, D. *et al*. Structural basis for PPAR partial or full activation revealed by a novel ligand binding mode. *Sci. Rep*. **6**, 34792; doi: 10.1038/srep34792 (2016).

## Supplementary Material

Supplementary Information

## Figures and Tables

**Table 1:**
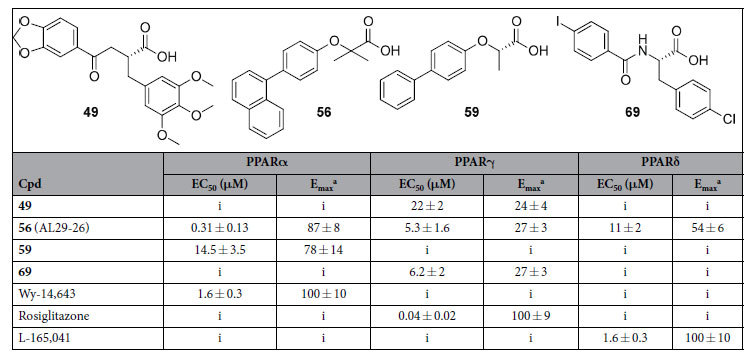
Activity of the Tested VS Hits in Cell-Based Transactivation Assays.

^a^Efficacy values were calculated as the percentage of the maximum obtained fold induction with the reference compounds. i = inactive at tested concentrations.

**Table 2:** Data collection and refinement statistics.

	PPARγ/AL29-26	PPARα/AL29-26
Data collection		
Space group	*C2*	*P4*_*1*_*2*_*1*_*2*
Cell dimension a, b, c (Å)	93.40, 60.79, 119.00	63.65, 63.65, 126.00
α, β, γ (°)	90, 103.4,90	90, 90, 90
X-ray source	synchrotron (ESRF)	synchrotron (ESRF)
N. of molecules in the A.U.	2	1
Wavelenght (Å)	0.873	0.873
Resolution (Å)	50–2.00	50–1.83
Unique reflections	23,665	43,099
Completeness (%)	98 (97)	100 (100)
R_merge_	5.9 (64.6)	7.1 (51.8)
I/σ(I)	10.1 (1.2	22.7 (5.0)
Refinement		
R_cryst_	22.5	21.1
R_free_	26.8	26.2
RMSD bond	0.009	0.7
RMSD angle	1.27	1.09
No. of residues		
Molecule A	251	262
Molecule B	251	
Ligands	2	1
Waters	187	220
Wilson B (Å^2^)	45.2	30.0
PDB entry	5HZC	5HYK

Values in parentheses are for the outer resolution shell.
