# Supplementary material for: Structural basis for PPAR partial or full activation revealed by a novel ligand binding mode
Source: Sci Rep. 2016 Oct 6;6:34792. doi: 10.1038/srep34792 (PMC5052532; doi:10.1038/srep34792)
Supplement: Supplementary Information [file srep34792-s1.pdf]

Structural basis for PPAR partial or full activation revealed by a novel ligand binding mode

Davide Capelli[1,5], Carmen Cerchia[2,5], Roberta Montanari[1], Fulvio Loiodice[3], Paolo Tortorella[3], Antonio Laghezza[3], Laura Cervoni[4], Giorgio Pochetti[1,*] and Antonio Lavecchia[2,*]
[1]Istituto di Cristallografia, Consiglio Nazionale delle Ricerche, Via Salaria Km. 29,300, 00015 Monterotondo Stazione, Roma, Italy
[2]Dipartimento di Farmacia, Università degli Studi di Napoli, Via Montesano 49, 80131 Napoli, Italy
[3]Dipartimento di Farmacia-Scienze del Farmaco, Università degli Studi di Bari "Aldo Moro", Via E.Orabona 4, 70126 Bari, Italy
[4]Dipartimento di Scienze Biochimiche "A. Rossi Fanelli", Università di Roma "La Sapienza", Piazzale A. Moro 5, 00185 Roma, Italy
[5]Co-first author
*Correspondence: giorgio.pochetti@ic.cnr.it (G.P.), antonio.lavecchia@unina.it (A.L.)

Supporting Information

Table S1. List of crystal structures of PPARγ in complex with partial agonists used for this study.

PDB code	Ligand code	Ligand name	Resolution (Å)	Reference
2FVJ	RO0	1-(3,4-dimethoxybenzyl)-6,7-dimethoxy-4-{[4-(2-methoxyphenyl)piperidin-1-yl]methyl}isoquinoline	1.99	1
2G0G	SP0	3-fluoro-n-[1-(4-fluorophenyl)-3-(2-thienyl)-1h-pyrazol-5-yl]benzenesulfonamide	2.54	2
2G0H	SP3	N-[1-(4-fluorophenyl)-3-(2-thienyl)-1h-pyrazol-5-yl]-3,5-bis(trifluoromethyl)benzenesulfonamide	2.3	2
2I4P	DRH	(2S)-2-(4-{2-[1,3-benzoxazol-2-yl(heptyl)amino]ethyl}phenoxy)-2-methylbutanoic acid	2.1	3
2I4Z	DRH	(2S)-2-(4-{2-[1,3-benzoxazol-2-yl(heptyl)amino]ethyl}phenoxy)-2-methylbutanoic acid	2.25	3
2P4Y	C03	(2R)-2-(4-chloro-3-{[3-(6-methoxy-1,2-benzisoxazol-3-yl)-2-methyl-6-(trifluoromethoxy)-1h-indol-1-yl]methyl}phenoxy)propanoic acid	2.25	4
2Q5P	241	(2S)-2-(3-{[1-(4-methoxybenzoyl)-2-methyl-5-	2.3	5

		(trifluoromethoxy)-1h-indol-3-yl]methyl}phenoxy)propanoic acid		
2Q5S	NZA	5-chloro-1-(4-chlorobenzyl)-3-(phenylthio)-1H-indole-2-carboxylic acid	2.05	[5]
2Q61	SF1	1-benzyl-5-chloro-3-(phenylthio)-1H-indole-2-carboxylic acid	2.2	[5]
2Q6R	SF2	5-chloro-1-(3-methoxybenzyl)-3-(phenylthio)-1H-indole-2-carboxylic acid	2.41	[5]
2Q6S	PLB	2-[(2,4-dichlorobenzoyl)amino]-5-(pyrimidin-2-yloxy)benzoic acid	2.4	[5]
2YFE	YFE	Amorfrutin 1	2	[6]
3B1M	KRC	(9AS)-8-acetyl-N-[(2-ethylnaphthalen-1-yl)methyl]-1,7-dihydroxy-3-methoxy-9Amethyl- 9-oxo-9,9A -dihydrodibenzo[B,D]furan-4-carboxamide	1.6	[7]
3CDP	YRG	(2S)-2-(4-chlorophenoxy)-3-phenylpropanoic acid	2.8	[8]
3D6D	LRG	(2S)-2-(biphenyl-4-yloxy)-3-phenylpropanoic acid	2.4	[9]
3FUR	Z12	2,4-dichloro-N-[3,5-dichloro-4-(quinolin-3-yloxy)phenyl]benzenesulfonamide	2.3	[10]
3H0A	D30	[(4-{[2-(pent-2-yn-1-yloxy)-4-{[4-(trifluoromethyl)phenoxy]methyl}phenyl]sulfanyl}-5,6,7,8-tetrahydronaphthalen-1-yl)oxy]acetic acid	2.1	[11]
3K8S	Z27	2-chloro-N-{3-chloro-4-[(5-chloro-1,3-benzothiazol-2-yl)sulfanyl]phenyl}-4-(trifluoromethyl)benzenesulfonamide	2.55	[12]
3LMP	CEK	(9AS)-8-acetyl-1,7-dihydroxy-3-methoxy-9A-methyl-N-(1-naphthylmethyl)-9-oxo-9,9A-dihydrodibenzo[B,D]furan-4-carboxamide	1.9	[13]
3OSI	XDH	4,4’-propane-2,2-diylbis(2,6-dichlorophenol)	2.7	[14]
3OSW	XDI	4,4’-propane-2,2-diylbis(2,6-dibromophenol)	2.55	[14]
3PBA	ZXG	2,6-dibromo-4-[2-(3,5-dibromo-4-hydroxyphenyl)propan-2-yl]phenyl hydrogen sulfate	2.3	[15]
4A4V	YFD	Amorfrutin 2	2	[16]
4A4W	YFB	Amorfrutin B	2	[16]
4PRG	072	(±)(2S,5S)-3-(4-(4-carboxyphenyl)butyl)-2-heptyl-4-	2.9	[17]

	oxo-5-thiazolidine

References

1. Burgermeister, E.; Schnoebelen, A.; Flament, A.; Benz, J.; Stihle, M.; Gsell, B.; Rufer, A.; Ruf, A.; Kuhn, B.; Marki, H. P.; Mizrahi, J.; Sebokova, E.; Niesor, E.; Meyer, M. A novel partial agonist of peroxisome proliferator-activated receptor-gamma (PPARgamma) recruits PPARgamma-coactivator-1alpha, prevents triglyceride accumulation, and potentiates insulin signaling in vitro. *Mol Endocrinol* **2006,** 20, 809-30.

2. Lu, I. L.; Huang, C. F.; Peng, Y. H.; Lin, Y. T.; Hsieh, H. P.; Chen, C. T.; Lien, T. W.; Lee, H. J.; Mahindroo, N.; Prakash, E.; Yueh, A.; Chen, H. Y.; Goparaju, C. M.; Chen, X.; Liao, C. C.; Chao, Y. S.; Hsu, J. T.; Wu, S. Y. Structure-based drug design of a novel family of PPARgamma partial agonists: virtual screening, X-ray crystallography, and in vitro/in vivo biological activities. *J Med Chem* **2006,** 49, 2703-12.

3. Pochetti, G.; Godio, C.; Mitro, N.; Caruso, D.; Galmozzi, A.; Scurati, S.; Loiodice, F.; Fracchiolla, G.; Tortorella, P.; Laghezza, A.; Lavecchia, A.; Novellino, E.; Mazza, F.; Crestani, M. Insights into the Mechanism of Partial Agonism: CRYSTAL STRUCTURES OF THE PEROXISOME PROLIFERATOR-ACTIVATED RECEPTOR γ LIGAND-BINDING DOMAIN IN THE COMPLEX WITH TWO ENANTIOMERIC LIGANDS. *Journal of Biological Chemistry* **2007,** 282, 17314-17324.

4. Einstein, M.; Akiyama, T. E.; Castriota, G. A.; Wang, C. F.; McKeever, B.; Mosley, R. T.; Becker, J. W.; Moller, D. E.; Meinke, P. T.; Wood, H. B.; Berger, J. P. The Differential Interactions of Peroxisome Proliferator-Activated Receptor γ Ligands with Tyr473 Is a Physical Basis for Their Unique Biological Activities. *Molecular Pharmacology* **2008,** 73, 62-74.

5. Bruning, J. B.; Chalmers, M. J.; Prasad, S.; Busby, S. A.; Kamenecka, T. M.; He, Y.; Nettles, K. W.; Griffin, P. R. Partial Agonists Activate PPARγ Using a Helix 12 Independent Mechanism. *Structure* **2007,** 15, 1258-1271.

6. Weidner, C.; de Groot, J. C.; Prasad, A.; Freiwald, A.; Quedenau, C.; Kliem, M.; Witzke, A.; Kodelja, V.; Han, C. T.; Giegold, S.; Baumann, M.; Klebl, B.; Siems, K.; Muller-Kuhrt, L.; Schurmann, A.; Schuler, R.; Pfeiffer, A. F.; Schroeder, F. C.; Bussow, K.; Sauer, S. Amorfrutins are potent antidiabetic dietary natural products. *Proc Natl Acad Sci U S A* **2012,** 109, 7257-62.

7. Wakabayashi, K.; Hayashi, S.; Matsui, Y.; Matsumoto, T.; Furukawa, A.; Kuroha, M.; Tanaka, N.; Inaba, T.; Kanda, S.; Tanaka, J.; Okuyama, R.; Wakimoto, S.; Ogata, T.; Araki, K.; Ohsumi, J. Pharmacology and in vitro profiling of a novel peroxisome proliferator-activated receptor gamma ligand, Cerco-A. *Biol Pharm Bull* **2011,** 34, 1094-104.

8. Fracchiolla, G.; Laghezza, A.; Piemontese, L.; Parente, M.; Lavecchia, A.; Pochetti, G.; Montanari, R.; Di Giovanni, C.; Carbonara, G.; Tortorella, P.; Novellino, E.; Loiodice, F. Synthesis, biological evaluation and molecular investigation of fluorinated peroxisome proliferator-activated receptors alpha/gamma dual agonists. *Bioorganic & medicinal chemistry* **2012,** 20, 2141-51.

9. Montanari, R.; Saccoccia, F.; Scotti, E.; Crestani, M.; Godio, C.; Gilardi, F.; Loiodice, F.; Fracchiolla, G.; Laghezza, A.; Tortorella, P.; Lavecchia, A.; Novellino, E.; Mazza, F.; Aschi, M.; Pochetti, G. Crystal Structure of the Peroxisome Proliferator-Activated Receptor γ (PPARγ) Ligand Binding Domain Complexed with a Novel Partial Agonist: A New Region of the Hydrophobic Pocket Could Be Exploited for Drug Design. *Journal of Medicinal Chemistry* **2008,** 51, 7768-7776.

10. Motani, A.; Wang, Z.; Weiszmann, J.; McGee, L. R.; Lee, G.; Liu, Q.; Staunton, J.; Fang, Z.; Fuentes, H.; Lindstrom, M.; Liu, J.; Biermann, D. H.; Jaen, J.; Walker, N. P.; Learned, R. M.; Chen, J. L.; Li, Y. INT131: a selective modulator of PPAR gamma. *J Mol Biol* **2009,** 386, 1301-11.

11. Connors, R. V.; Wang, Z.; Harrison, M.; Zhang, A.; Wanska, M.; Hiscock, S.; Fox, B.; Dore, M.; Labelle, M.; Sudom, A.; Johnstone, S.; Liu, J.; Walker, N. P.; Chai, A.; Siegler, K.; Li, Y.; Coward, P. Identification of a PPARdelta agonist with partial agonistic activity on PPARgamma. *Bioorg Med Chem Lett* **2009,** 19, 3550-4.

12. Li, Y.; Wang, Z.; Furukawa, N.; Escaron, P.; Weiszmann, J.; Lee, G.; Lindstrom, M.; Liu, J.; Liu, X.; Xu, H.; Plotnikova, O.; Prasad, V.; Walker, N.; Learned, R. M.; Chen, J. L. T2384, a novel antidiabetic agent with unique peroxisome proliferator-activated receptor gamma binding properties. *J Biol Chem* **2008,** 283, 9168-76.

13. Furukawa, A.; Arita, T.; Satoh, S.; Wakabayashi, K.; Hayashi, S.; Matsui, Y.; Araki, K.; Kuroha, M.; Ohsumi, J. Discovery of a novel selective PPARgamma modulator from (-)-Cercosporamide derivatives. *Bioorg Med Chem Lett* **2010,** 20, 2095-8.

14. Riu, A.; Grimaldi, M.; le Maire, A.; Bey, G.; Phillips, K.; Boulahtouf, A.; Perdu, E.; Zalko, D.; Bourguet, W.; Balaguer, P. Peroxisome Proliferator-Activated Receptor γ Is a Target for Halogenated Analogs of Bisphenol A. *Environ Health Persp* **2011,** 119, 1227-1232.

15. Riu, A.; le Maire, A.; Grimaldi, M.; Audebert, M.; Hillenweck, A.; Bourguet, W.; Balaguer, P.; Zalko, D. Characterization of Novel Ligands of ERα, Erβ, and PPARγ: The Case of Halogenated Bisphenol A and Their Conjugated Metabolites. *Toxicological Sciences* **2011,** 122, 372-382.

16. de Groot, J. C.; Weidner, C.; Krausze, J.; Kawamoto, K.; Schroeder, F. C.; Sauer, S.; Bussow, K. Structural characterization of amorfrutins bound to the peroxisome proliferator-activated receptor gamma. *J Med Chem* **2013,** 56, 1535-43.

17. Oberfield, J. L.; Collins, J. L.; Holmes, C. P.; Goreham, D. M.; Cooper, J. P.; Cobb, J. E.; Lenhard, J. M.; Hull-Ryde, E. A.; Mohr, C. P.; Blanchard, S. G.; Parks, D. J.; Moore, L. B.; Lehmann, J. M.; Plunket, K.; Miller, A. B.; Milburn, M. V.; Kliewer, S. A.; Willson, T. M. A peroxisome proliferator-activated receptor gamma ligand inhibits adipocyte differentiation. *Proc Natl Acad Sci U S A* **1999,** 96, 6102-6.

Table S2. Compounds identified as hits against PPARγ from the SBVS and their activity in cell-based transactivation assays.

		PPARα		PPARγ		PPARδ		
Cpd	2D Structure	EC_{50} (μM)	E_{max} [a]	EC_{50} (μM)	E_{max} [a]	EC_{50} (μM)	E_{max} [a]	XP score
1	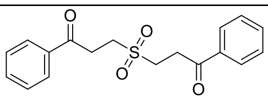	i	i	i	i	i	i	-6.47
2	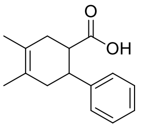	i	i	i	i	i	i	-6.29
3	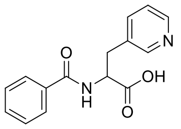	i	i	i	i	i	i	-6.04
4	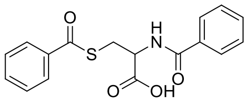	i	i	i	i	i	i	-7.28
5	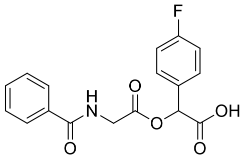	i	i	i	i	i	i	-6.41
6	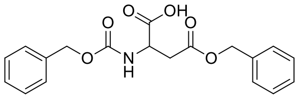	4.4 ± 2.4	124 ± 12	14.8 ± 0.9	10 ± 6	i	i	-8.74
7	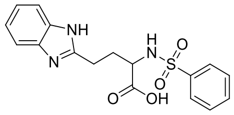	i	i	i	i	i	i	-7.68
8	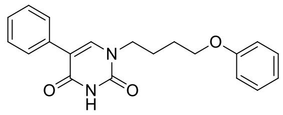	i	i	i	i	i	i	-8.54
9	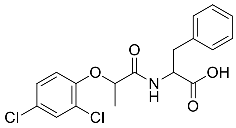	i	i	i	i	i	i	-8.98
10	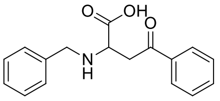	i	i	i	i	i	i	-8.70
11	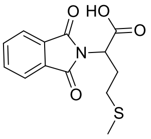	i	i	i	i	i	i	-6.76
12	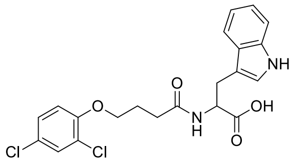	i	i	i	i	i	i	-5.33
13	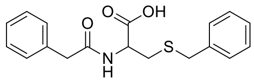	2.55 ± 0.35	148 ± 22	10.9 ± 2.4	16 ± 7	i	i	-8.47
14	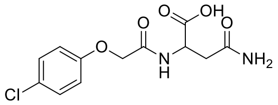	i	i	i	i	i	i	-6.95

15	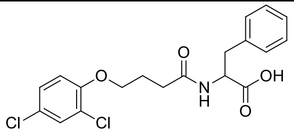	i	i	i	i	i	i	-9.36
16	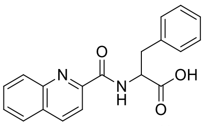	i	i	i	i	i	i	-8.70
17	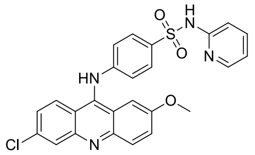	i	i	i	i	i	i	-6.83
18	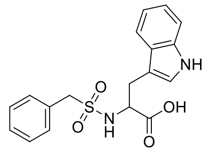	i	i	i	i	i	i	-5.88
19	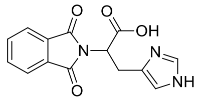	i	i	i	i	i	i	-8.53
20	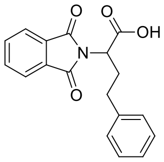	i	i	i	i	i	i	-8.25
21	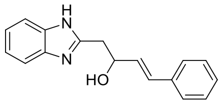	i	i	i	i	i	i	-7.43
22	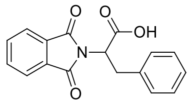	i	i	i	i	i	i	-8.24
23	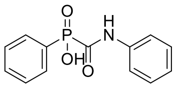	i	i	i	i	i	i	-6.87
24	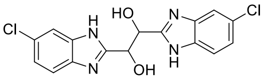	i	i	i	i	i	i	-8.01
25	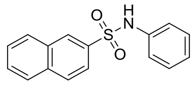	i	i	i	i	i	i	-5.96
26	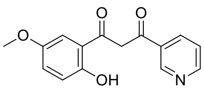	i	i	i	i	i	i	-7.11
27	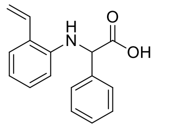	i	i	i	i	i	i	-7.36
28	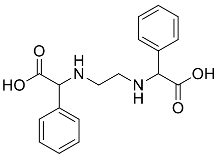	i	i	i	i	i	i	-6.66
29	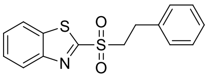	i	i	i	i	i	i	-6.47
30	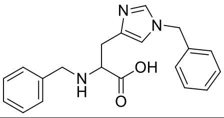	i	i	i	i	i	i	-6.71

31	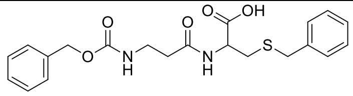	i	i	i	i	i	i	-10.16
32	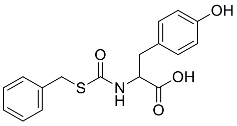	i	i	i	i	i	i	-8.54
33	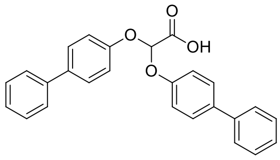	1.74 ± 0.05	42 ± 2	0.91 ± 0.34	53 ± 5	i	i	-7.16
34	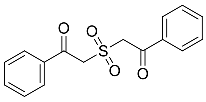	i	i	i	i	i	i	-6.17
35	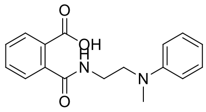	i	i	i	i	i	i	-7.68
36	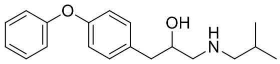	i	i	i	i	i	i	-7.31
37	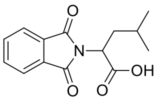	i	i	i	i	i	i	-7.27
38	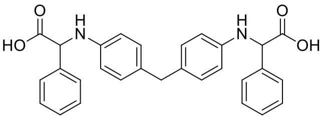	2.55 ± 0.21	32 ± 1	11 ± 1	11 ± 4	8.4 ± 2	16 ± 1	-3.11
39	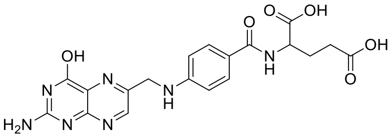	i	i	i	i	i	i	-8.28
40	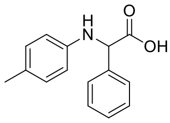	18.85 ± 2.90	37 ± 12	i	i	i	i	-5.19
41	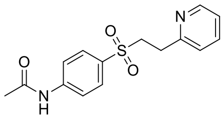	i	i	i	i	i	i	-5.97
42	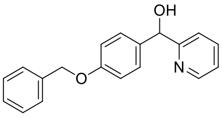	i	i	i	i	i	i	-6.95
43	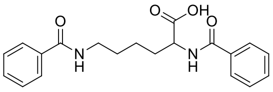	i	i	i	i	i	i	-7.17
44	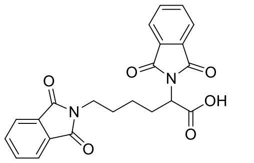	i	i	i	i	i	i	-7.95
Wy-14,643		1.6 ± 0.3	100 ± 10	i	i	i	i	
Rosiglitazone		i	i	0.04 ± 0.02	100 ± 9	i	i	
L-165,041		i	i	i	i	1.6 ± 0.3	100 ± 10	

[a] Efficacy values were calculated as the percentage of the maximum obtained fold induction with the reference compounds. i = inactive at tested concentrations.

Table S3. Structures of hits selected by substructure search.

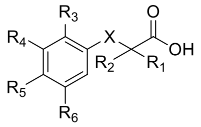

45-59

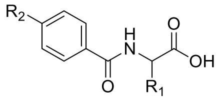

60-69

Cpd	X	R_1	R_2	R_3	R_4	R_5	R_6
45	CH_2	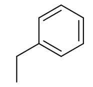	H	H	H	H	H
46	CH_2	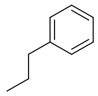	H	COOH	H	H	H
47	CH_2	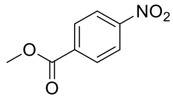	H	H	H	H	H
48	CH_2	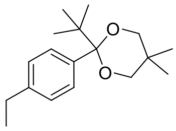	H	H	H	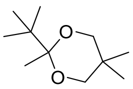	H
49	CH_2	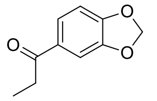	H	H	OCH_3	OCH_3	OCH_3
50	CH_2	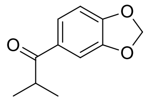	H	H	OCH_3	OCH_3	OCH_3
51	CH_2	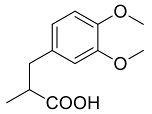	H	H	H	OCH_3	OCH_3
52	CH_2	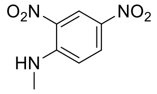	H	H	H	H	H
53	CHOH	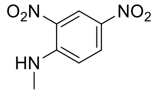	H	H	H	H	H
54	CO	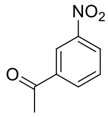	H	H	NO_2	H	H
55	O	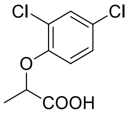	H	Cl	H	Cl	H
56	O	CH_3	CH_3	H	H	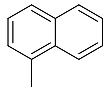	H
57	O	H	H	H	H	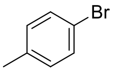	H
58	O	H	H	H	H	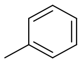	H
59	O	CH_3	H	H	H	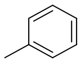	H
60	-	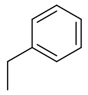	H	-	-	-	-

61	-	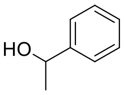	H	-	-	-	-
62	-	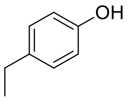	H	-	-	-	-
63	-	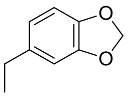	H	-	-	-	-
64	-	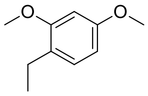	H	-	-	-	-
65	-	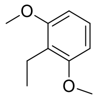	H	-	-	-	-
66	-	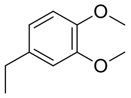	H	-	-	-	-
67	-	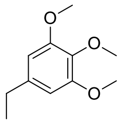	H	-	-	-	-
68	-	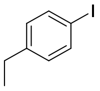	I	-	-	-	-
69	-	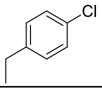	I	-	-	-	-

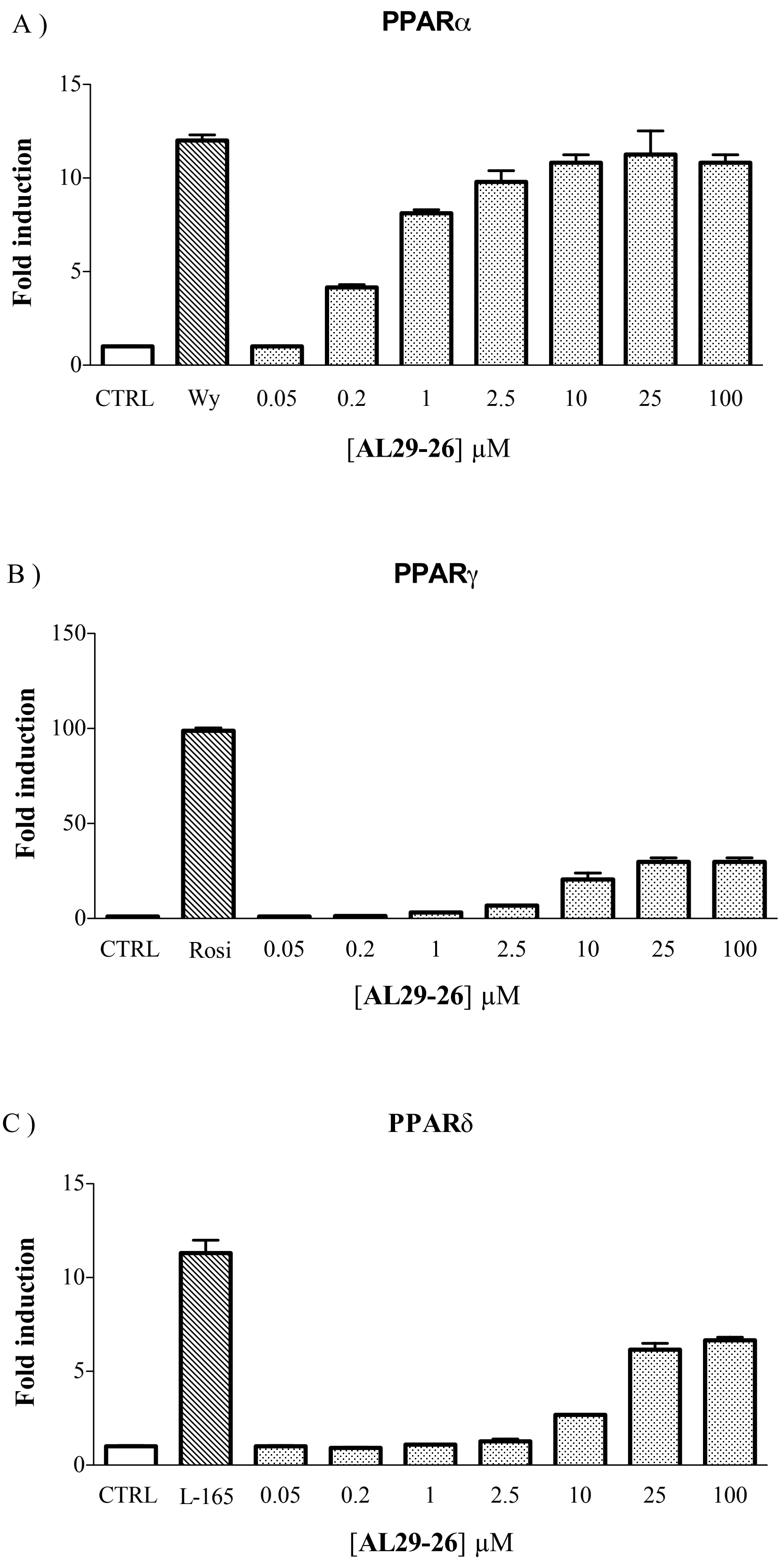

Figure S1. Transactivation assay on PPARα (A), PPARγ (B) and PPARδ (C) of AL29-26 in HepG2 cells. Data are expressed as fold change compared with vehicle-treated cells (DMSO, 0.1%) and represent the mean of assays performed in triplicate

± SEM. Reference compounds: Wy-14,643 (10 μM) rosiglitazone (2 μM) and L-165,041 (2 μM).

A)

PPARα

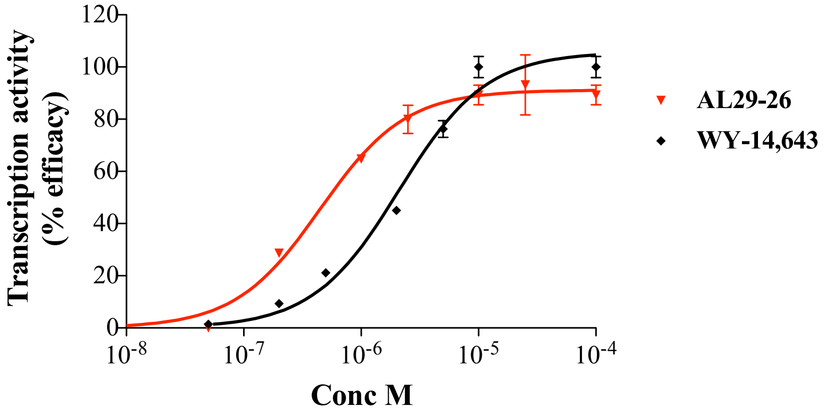

B)

PPARγ

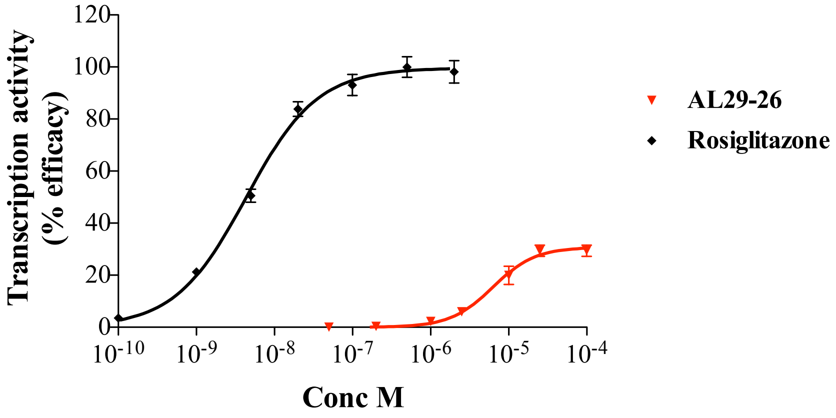

C)

PPARδ

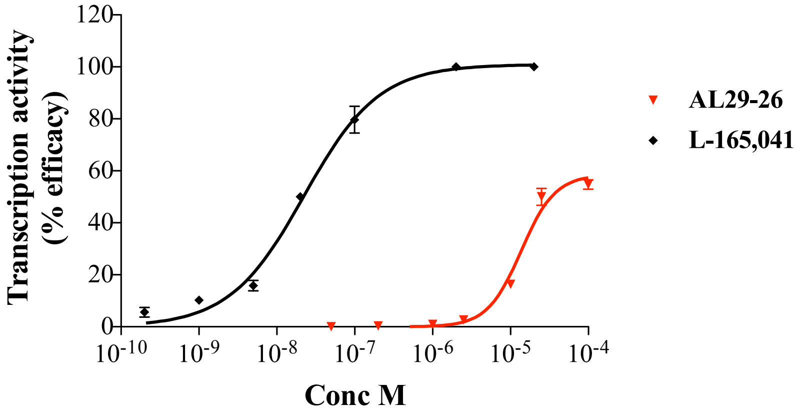

Fig. S2. Activation of GAL4-PPAR ligand binding domain chimeras in transiently transfected HepG2 cells by AL29-26. Dose–response curves are shown for human

PPARα (A), PPARγ (B) and PPARδ (C). The curves are expressed as percentage of maximum effect obtained by reference compounds and represent the mean of assays performed in triplicate ± SEM.

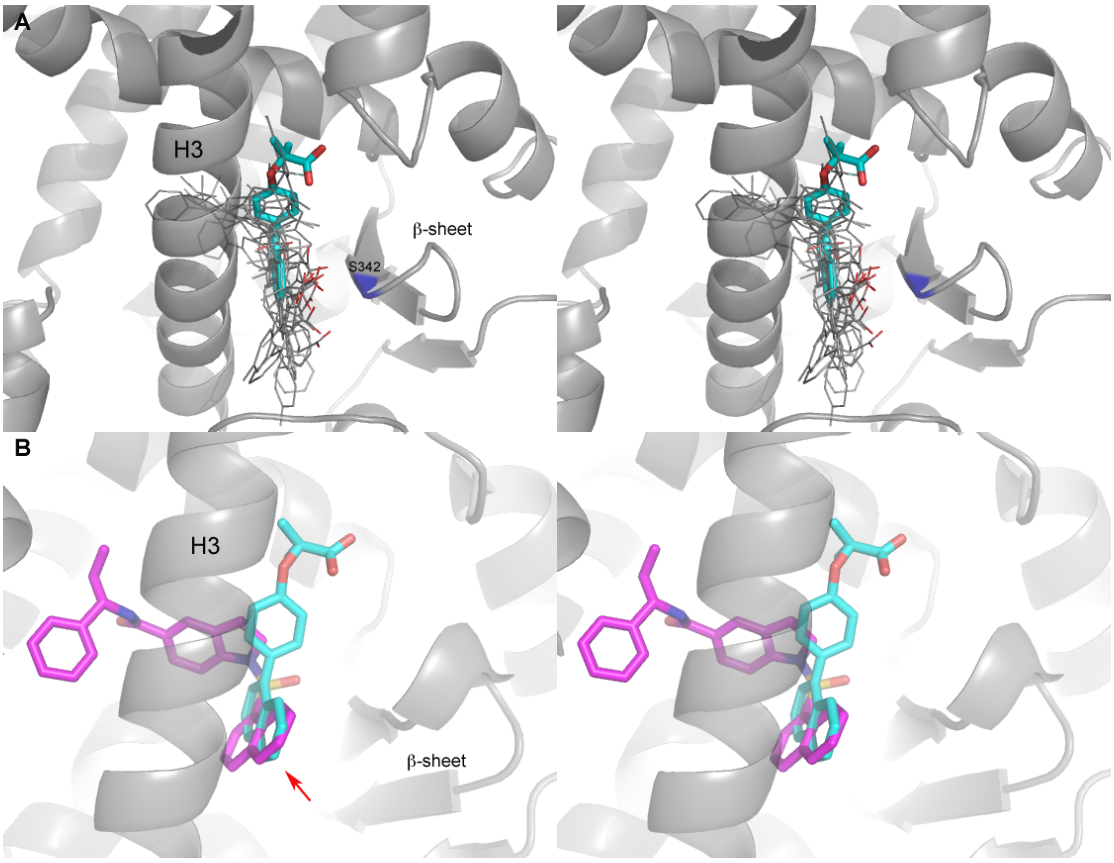

Figure S3. Comparison of PPARγ complexes (stereo view)

(A) Superposition of PPARγ complexes (gray) with known partial agonists (pdb codes: 3D6D, 4PVU, 4PWL, 4JL4, 4JAZ, 4E4K, 2Q5P, 2Q6S, 2Q5S, 4E4Q, 5F9B) onto the PPARγ complex with AL29-26 (cyan). The carboxylate groups are depicted in red, the residue S342 in blue.

(B) Superposition of SR2067 (magenta) (pdb code 4R06) onto the PPARγ complex with AL29-26 (cyan). The red arrow indicates the naphthalene groups of the two ligands.

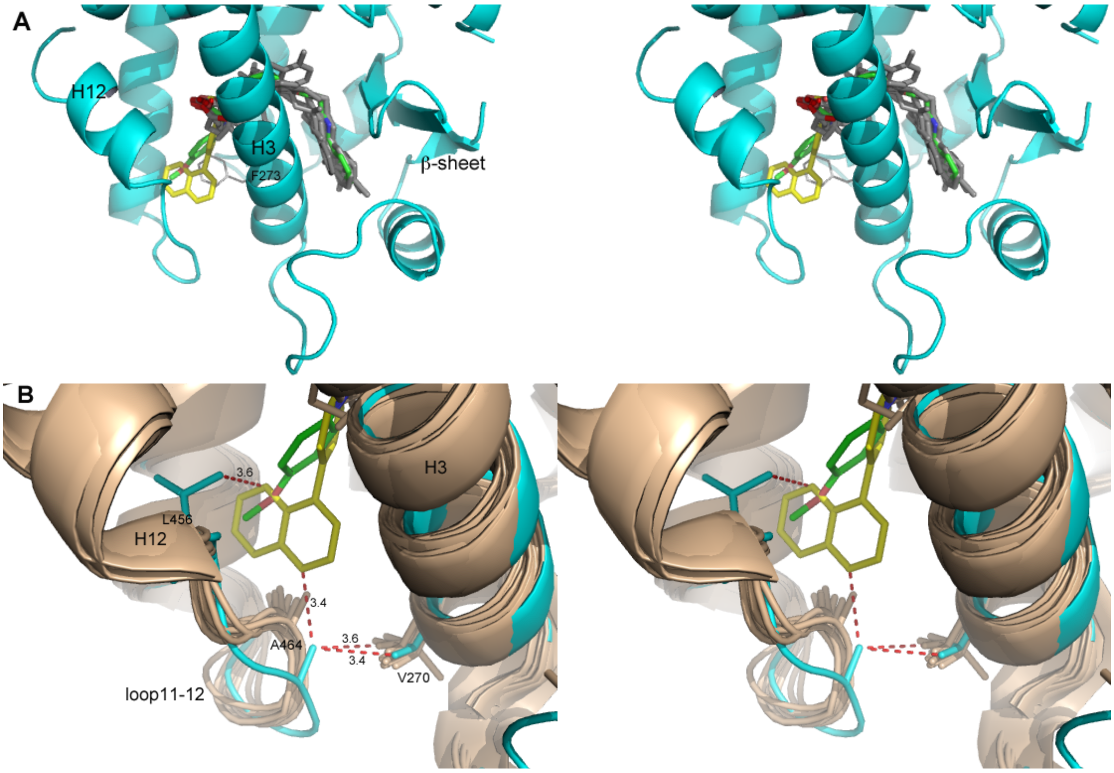

Figure S4. Comparison of PPARα complexes

(A) Superposition of PPARα complexes (gray) with known partial agonists (pdb codes: 2REW, 4BCR, 1K7L, 3SP6, 3FEI, 2GTK, 3G8I, 3ET1, 3KDT, 1I7G) onto the PPARα complex with AL29-26 (ligand yellow, protein cyan). The ligand BMS-631707 (PDB code 2REW) is shown in green. The “closed” (trans) conformation of F273 side-chain is also shown (gray).

(B) New conformation of the loop 11-12 in the PPARα/AL29-26 complex: superposition of the loops 11-12 of known PPARα structures (light-brown) (same pdb codes of Figure 5A) with that of PPARα/AL29-26 (ligand yellow, protein cyan). The ligand BMS-631707 (PDB code 2REW) is shown in green. Additional vdW interactions realized by AL29-26 are shown as red dashed lines.

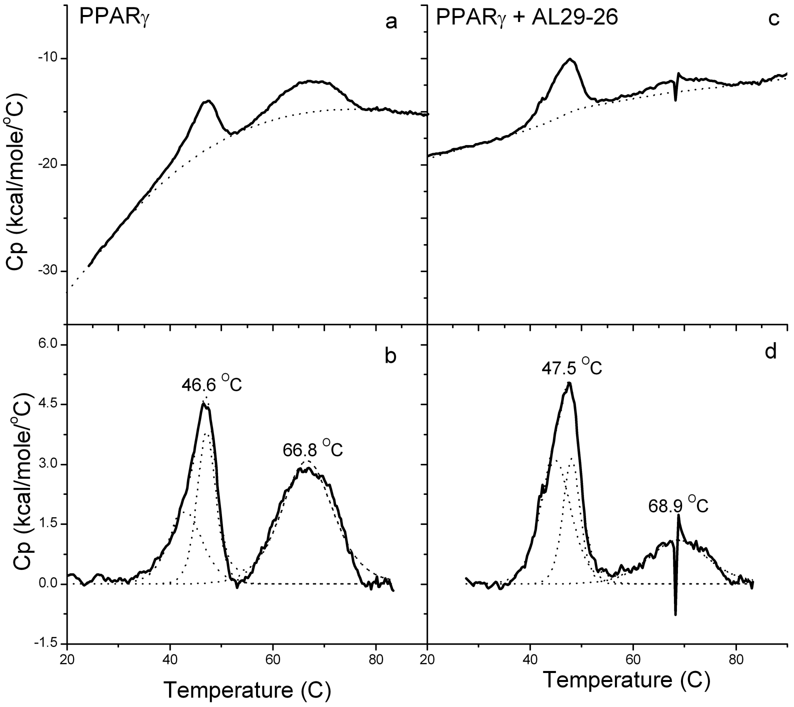

Figure S5. DSC thermograms of PPARγ

(A) Experimental curve (solid line) and base line (dotted line) of PPARγ 0.4 mg/ml in buffer Hepes 20 mM, pH8.0, TCEP 1 mM (scan speed 60 °C/h).

(B) Experimental curve after subtraction of the base line and deconvolution of the excess heat capacity function into three non-two-state transitions (dotted lines).

(C) Experimental curve (solid line) and base line (dotted line) of PPARγ 0.4 mg/ml with AL29-26 20 μM, in buffer Hepes 20 mM, pH8.0, TCEP 1 mM (scan speed 60 °C/h).

(D) Experimental curve after subtraction of the base line and deconvolution of the excess heat capacity function into three non-two-state transitions (dotted lines).

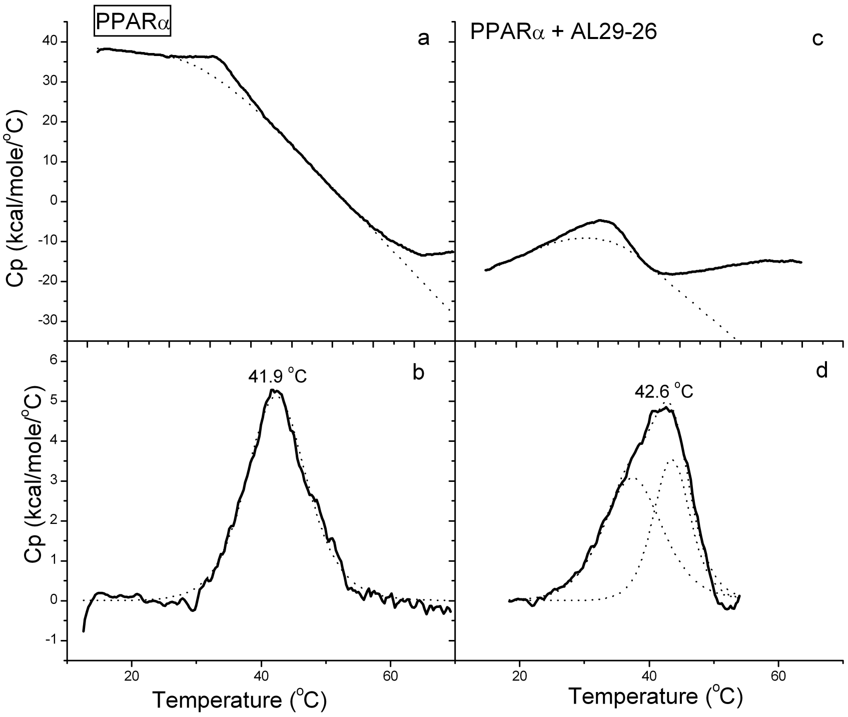

Figure S6. DSC thermograms of PPARα

(A) Experimental curve (solid line) and base line (dotted line) of PPARα 0.4 mg/ml in buffer Hepes 20 mM, pH8.0, TCEP 1 mM (scan speed 60 °C/h).

(B) Experimental curve after subtraction of the base line and deconvolution of the excess heat capacity function into one two-state transition (dotted lines).

(C) Experimental curve (solid line) and base line (dotted line) of PPARα 0.4 mg/ml with AL29-26 20 μM, in buffer Hepes 20 mM, pH8.0, TCEP 1 mM (scan speed 60 °C/h).

(D) Experimental curve after subtraction of the base line and deconvolution of the excess heat capacity function into two two-state transitions (dotted lines).
